# Identification of Rare Loss-of-Function Genetic Variation Regulating Body Fat Distribution

**DOI:** 10.1210/clinem/dgab877

**Published:** 2021-12-07

**Authors:** Mine Koprulu, Yajie Zhao, Eleanor Wheeler, Liang Dong, Nuno Rocha, Chen Li, John D Griffin, Satish Patel, Marcel Van de Streek, Craig A Glastonbury, Isobel D Stewart, Felix R Day, Jian’an Luan, Nicholas Bowker, Laura B L Wittemans, Nicola D Kerrison, Lina Cai, Debora M E Lucarelli, Inês Barroso, Mark I McCarthy, Robert A Scott, Vladimir Saudek, Kerrin S Small, Nicholas J Wareham, Robert K Semple, John R B Perry, Stephen O’Rahilly, Luca A Lotta, Claudia Langenberg, David B Savage

**Affiliations:** 1 MRC Epidemiology Unit, University of Cambridge School of Clinical Medicine, Institute of Metabolic Science, Cambridge, CB2 0QQ, UK; 2 University of Cambridge Metabolic Research Laboratories, Wellcome Trust-MRC Institute of Metabolic Science, Cambridge, CB2 0QQ, UK; 3 Internal Medicine Research Unit, Pfizer Worldwide Research, Development, and Medical, Cambridge, Massachusetts 02139, USA; 4 Department of Twin Research and Genetic Epidemiology, King’s College London, St Thomas’ Campus, London, SE1 7EH, UK; 5 BenevolentAI Limited, London, W1T 5HD, UK; 6 Big Data Institute at the Li Ka Shing Centre for Health Information and Discovery, University of Oxford, Oxford, OX3 7LF, UK; 7 Nuffield Department of Women’s and Reproductive Health, Medical Sciences Division, University of Oxford, Oxford, OX3 9DU, UK; 8 Exeter Centre of Excellence for Diabetes Research (EXCEED), Genetics of Complex Traits, University of Exeter Medical School, University of Exeter, Exeter, EX1 2HZ, UK; 9 Wellcome Centre for Human Genetics, University of Oxford, Oxford, OX3 7BN, UK; 10 Centre for Cardiovascular Science, University of Edinburgh, Edinburgh, EH16 4TJ, UK; 11 Computational Medicine, Berlin Institute of Health at Charité–Universitätsmedizin Berlin, 10117 Berlin, Germany

**Keywords:** fat distribution, cardiometabolic risk, genetic variants, loss of function, UK Biobank

## Abstract

**Context:**

Biological and translational insights from large-scale, array-based genetic studies of fat distribution, a key determinant of metabolic health, have been limited by the difficulty in linking predominantly noncoding variants to specific gene targets. Rare coding variant analyses provide greater confidence that a specific gene is involved, but do not necessarily indicate whether gain or loss of function (LoF) would be of most therapeutic benefit.

**Objective:**

This work aimed to identify genes/proteins involved in determining fat distribution.

**Methods:**

We combined the power of genome-wide analysis of array-based rare, nonsynonymous variants in 450 562 individuals in the UK Biobank with exome-sequence-based rare LoF gene burden testing in 184 246 individuals.

**Results:**

The data indicate that the LoF of 4 genes (*PLIN1* [LoF variants, *P* = 5.86 × 10^–7^], *INSR* [LoF variants, *P* = 6.21 × 10^–7^], *ACVR1C* [LoF + moderate impact variants, *P* = 1.68 × 10^–7^; moderate impact variants, *P* = 4.57 × 10^–7^], and *PDE3B* [LoF variants, *P* = 1.41 × 10^–6^]) is associated with a beneficial effect on body mass index–adjusted waist-to-hip ratio and increased gluteofemoral fat mass, whereas LoF of *PLIN4* (LoF variants, *P* = 5.86 × 10^–7^ adversely affects these parameters. Phenotypic follow-up suggests that LoF of *PLIN1*, *PDE3B*, and *ACVR1C* favorably affects metabolic phenotypes (eg, triglycerides [TGs] and high-density lipoprotein [HDL] cholesterol concentrations) and reduces the risk of cardiovascular disease, whereas *PLIN4* LoF has adverse health consequences. *INSR* LoF is associated with lower TG and HDL levels but may increase the risk of type 2 diabetes.

**Conclusion:**

This study robustly implicates these genes in the regulation of fat distribution, providing new and in some cases somewhat counterintuitive insight into the potential consequences of targeting these molecules therapeutically.

Fat distribution is a heritable trait, commonly estimated by the relative amounts of waist and hip fat (waist-to-hip ratio, WHR) for a given body size. Genetic mechanisms linked to either relatively lower gluteofemoral or higher abdominal fat or both have been shown to contribute to a greater WHR and its consistently adverse cardiometabolic consequences (1). Genome-wide array-based association studies have robustly identified many loci linked to WHR but thus far provided relatively limited biological and translational insights because of poor coverage of rare protein-coding variants and uncertainties connecting associated noncoding variants to functional genes (2, 3). Consequently, very few genes have been definitively linked to WHR and it is generally unknown whether a gain or loss of gene function (LoF) is likely to drive observed associations.

The low frequency of rare (minor allele frequency [MAF] < 0.5%, as defined by the 1000 Genomes Project) (4) functional variants may be a consequence of selective pressure acting against them as a result of sizable effects on the encoded protein. Previous studies have shown inverse relationships between allele frequency and effect size for complex traits (5). Rare variants that occurred recently are also likely to be in low linkage disequilibrium with nearby common variants, facilitating fine-mapping and identification of causal variants and genes (6). However, rare variants are difficult to impute (7) so their study requires large, homogeneous samples and direct genotyping. To date, the vast majority of studies have explored the contribution of common variants in relation to WHR including the largest meta-analysis of imputed genome-wide association studies, which included up to 694 649 individuals but identified only 2 variants at MAF 0.1% to 0.5% (3). The only other study that investigated the role of rare variants for WHR was a subsequent transethnic ExomeChip effort that identified 9 low-frequency or rare variants with a lowest MAF of 0.1% (8).

The contribution of the full spectrum of rare variants to WHR using sequence data has not been studied, yet has the potential to provide a more direct link between gene and phenotype, and to facilitate translation from gene identification to drug development. While the identification of coding variants in a specific gene clearly increases confidence in linking that particular gene to a trait, the effect of individual coding variants can still be very, or at least relatively, subtle. Individual variant testing, even using exome sequencing data in large populations, therefore still provides limited power and leaves residual uncertainty about the benefits of gain or LoF of a particular gene. Exome-wide scans of the gene-based burden of rare LoF variants have the potential to address this limitation (9-13). In this study, we use a “dual approach” that combines the power of large-scale genome-wide analysis of array-based rare, nonsynonymous variants with validation using data from exome sequencing as well as exome sequence–based rare gene burden testing to identify the putative function of variants, genes, and pathways regulating body shape and fat distribution (assessed by body mass index [BMI]-adjusted WHR [WHR_adjBMI_]) and to determine their effects on body composition and metabolic health.

## Results

**Figure 1.**
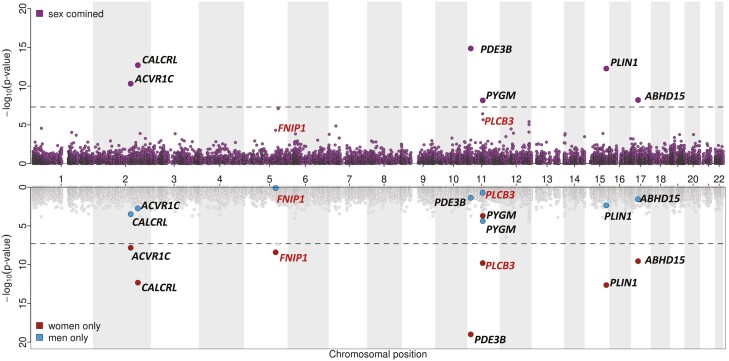
Miami plot for sex-combined and sex-specific single-marker association results for body mass index–adjusted waist-to-hip ratio (WHR_adjBMI_). Top: Manhattan plot representing results from the main, sex-combined genome-wide association studies for WHR_adjBMI_ for genotyped, rare nonsynonymous variants (MAF = 0.1%-0.5%, correlation and rare allele concordance > 0.9 when compared to the exome sequencing data). Gene annotations for the genome-wide significant variants from the main, sex-combined analyses are shown in black; gene annotations and significance from the main, sex-combined analyses for variants that were genome-wide significant in sex-specific analyses (women) only are shown in red. Bottom: Sex-specific significance of the variants highlighted previously.

A genome-wide analysis of directly genotyped, rare (MAF = 0.1%-0.5%) nonsynonymous variants associated with WHR_adjBMI_ at *P* less than 5 × 10^–8^ in 450 562 European ancestry individuals from the UK Biobank identified lead variants in *PLIN1* p.L90P (rs139271800, effect allele frequency [EAF] = 0.1%), *PDE3B* p.R783X (rs150090666, EAF = 0.1%), *ACVR1C* p.I195T (rs56188432, EAF = 0.2%), *CALCRL* p.L87P (rs61739909; EAF = 0.3%), *ABHD15* p.G147D (rs141385558; EAF = 0.2%), and *PYGM* p.R50X (rs116987552, EAF = 0.4%) (Fig. 1, Supplementary Table 1) (14). All variants other than *PYGM* p.R50X (rs116987552) have previously been reported to be associated with WHR_adjBMI_ (3, 15). We observed a correlation of 0.99 and minor allele concordance of 0.99 comparing genotyped to whole-exome sequenced (WES) rare (MAF = 0.1%-0.5%) nonsynonymous variants when testing the validity of rare, genotyped variants using exome-sequencing data from the overlapping samples (Supplementary Table 2) (14).

### Sex Differences in Genetic Effects of Rare Variants on Body Mass Index–adjusted Waist-to-Hip Ratio

Common variant analyses have provided evidence of differences in genetic associations with fat distribution between men and women (3). In line with this, we found evidence of statistically significant sex interactions, with stronger genetic effects in women, compared to men for all lead variants, except for *ACVR1C* p.I195T and *PYGM* p.R50X (Table 1). We therefore conducted sex-specific analyses that revealed 2 additional variants, *PLCB3* p.V806I (rs145502455, EAF = 0.4%) and *FNIP1* p.R518Q (rs115209326, EAF = 0.3%) to be genome significant in (*P* < 5 × 10^–8^) in women, with no effect in men (see Table 1 and Supplementary Table 1) (14). No variants reached genome-wide statistical significance in men only.

**Table 1.** Sex-stratified results for variants identified in joint and sex specific analyses of genotyped rare variants in UK Biobank

Variant	rsID	*P* sex interaction	*P* Women	*P* Men	β Women	β Men	SE Women	SE Men
***PLIN1* p.L90P**	rs139271800	4.07 × 10^–3^	1.90 × 10^–13^	4.50 × 10^–3^	–0.273	–0.126	0.039	0.042
***PDE3B* p.R783X**	rs150090666	5.66 × 10^–5^	1.00 × 10^–19^	4.10 × 10^–2^	–0.392	–0.102	0.042	0.048
***ACVR1C* p.I195T**	rs56188432	0.36 × 10^–1^	1.10 × 10^–8^	3.40 × 10^–4^	–0.16	–0.109	0.028	0.032
***CALCRL* p.L87P**	rs61739909	4.30 × 10^–3^	4.30 × 10^–13^	2.50 × 10^–3^	–0.171	–0.082	0.024	0.026
***ABHD15* p.G147D**	rs141385558	4.53 × 10^–3^	3.40 × 10^–10^	3.80 × 10^–2^	0.168	0.057	0.026	0.029
***PYGM* p.R50X**	rs116987552	0.36 × 10^–1^	2.90 × 10^–4^	5.40 × 10^–5^	0.079	0.095	0.021	0.024
***PLCB3* p.V806I**	rs145502455	2.34 × 10^–2^	1.60 × 10^–10^	0.20 × 10^–1^	0.126	0.029	0.021	0.024
***FNIP1* p.R518Q**	rs115209326	6.09 × 10^–4^	4.80 × 10^–9^	0.84 × 10^–1^	–0.128	–0.003	0.023	0.026

Unshaded rows present results discovered from the sex-combined analysis, and gray-shaded rows represent results from variants identified only in the sex-stratified (women) analysis.

Abbreviations: β Men, effect size in men; β Women, effect size in women; *P* Men, BOLT LMM *P* value in men; *P* Women, BOLT LMM *P* value in women.

### Genomic Context and Fine-Mapping Analyses

To identify variants responsible (causal) for the association with WHR_adjBMI_, we conducted systematic analyses of genomic context through fine-mapping of statistically decomposed signals at each locus to establish whether the identified rare nonsynonymous variants are likely to mechanistically contribute to variation in WHR_adjBMI_ (see “Materials and Methods”). We found strong statistical evidence (ie, posterior probability of casual association > 50%) that the rare nonsynonymous variants in *PLIN1*, *PDE3B*, *ACVR1C*, and *CALCRL* were causal for the association with WHR_adjBMI_ (Supplementary Note 1, Supplementary Table 3, and Supplementary Fig. 1) (14). Genomic context analyses did not support the causality of the identified rare lead variants in *ABHD15* or *PYGM* from the joint (sex-combined) analysis, and of *PLCB3* and *FNIP1* in the women-only analysis (Supplementary Note 2 and Supplementary Table 4) (14). Bioinformatic analysis of these variants strongly predicted that the *PDE3B* variant p.R783X would truncate *PDE3B* within the catalytic site, impairing *PDE3B* catalytic activity if expressed (Supplementary Note 3) (14), whereas predictions of the functional effect of the *PLIN1*, *ACVR1C*, and *CALCRL* variants were less conclusive (Supplementary Note 3) (14).

### Exome Sequenced–Based Burden Testing of Rare, Loss-of Function Variants

Next, we considered the genes identified in the single-variant analysis for exome sequence–based gene rare LoF and missense burden testing in 184 246 individuals in the UK Biobank (see “Materials and Methods,” Gene-based association testing) and found that *PLIN1*, *PDE3B*, *ACVR1C*, and *CALCRL* were all statistically significantly associated with lower WHR_adjBMI_ at a Bonferroni-corrected threshold (*P* < .0125). Predicted loss of function (pLoF) variants showed the most statistically significant association for *PLIN1* and *PDE3B*, moderate impact variants for *CALCRL*, and the combination of pLoF with moderate impact variants for *ACVR1C* (Supplementary Table 5) (14).

**Figure 2.**
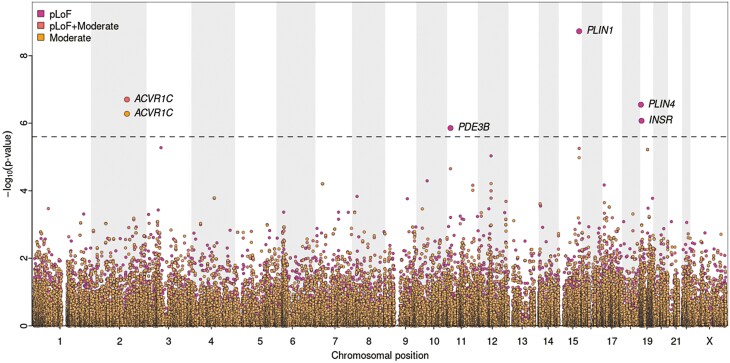
Gene-based association results. Gene-based exome-wide discovery results for body mass index–adjusted waist-to-hip ratio (WHR_adjBMI_). The horizontal dashed line represents the exome-wide significance threshold (*P* = 2.53 × 10^–6^).

To identify additional genes in which loss of function may regulate fat distribution, we extended this approach to a hypothesis free, exome-wide analysis for WHR_adjBMI_ using more stringent quality control (QC) parameters (see “Materials and Methods”). This identified *PLIN4* and *INSR* in at least one of the variant categories (see “Materials and Methods”), in addition to *PLIN1, ACVR1C* in the sex-combined analyses (*P* < 8.44 × 10^–7^; exome-wide threshold corrected for multiple testing, see “Materials and Methods”) (Fig. 2 and Supplementary Table 6) (14)*. PDE3B* also reached suggestive exome-wide statistical significance (*P* < 2.53 × 10^–6^) (see Fig. 2 and Supplementary Table 6) (14). All genes identified in the sex-combined analysis, including *PDE3B*, were statistically significant (*P* < 8.44 × 10^–7^) in the women-only analyses. No genes reached statistical significance (*P* < 8.44 × 10^–7^) in men-only analyses. *PLIN4*, *INSR*, and *PDE3B* all showed statistically significantly larger standardized effect sizes for women compared to men (*P* < .05) in gene-based analyses, in line with the single-marker results (Supplementary Table 7) (14).

While the joint effect of rare LoF variants in *PLIN4* (65 variants, 1065 carriers) was associated with a higher WHR_adjBMI_ (β = 0.16 [0.10-0.22], *P* = 5.86 × 10^–7^), the combination of rare LoF variants in *PLIN1* (31 variants, 393 carriers) was associated with a lower WHR_adjBMI_ (β = –0.27 [–0.17 to –0.36], *P* = 9.82 × 10^–9^) (see Supplementary Table 6) (14). The lead *PLIN1* LoF variant (*PLIN1* p.T338DfsX51, rs750619494) is predicted to result in a frameshift from amino acid 338 with a premature stop at amino acid 388, though it may well be subject to nonsense-mediated RNA decay. Several additional *PLIN1* variants are similarly expected to result in early truncations or nonsense-mediated RNA decay (Supplementary Table 8) (14). In either instance, these variants are expected to impair Plin1 interaction with Abhd5 and thus its regulation of adipose triglyceride lipase (Atgl) (16). In the case of *PLIN4* (p.Q372X, rs201581703), the variant list also included early frameshift/premature stop variants predicted to result in nonsense-mediated RNA decay.

**Figure 3.**
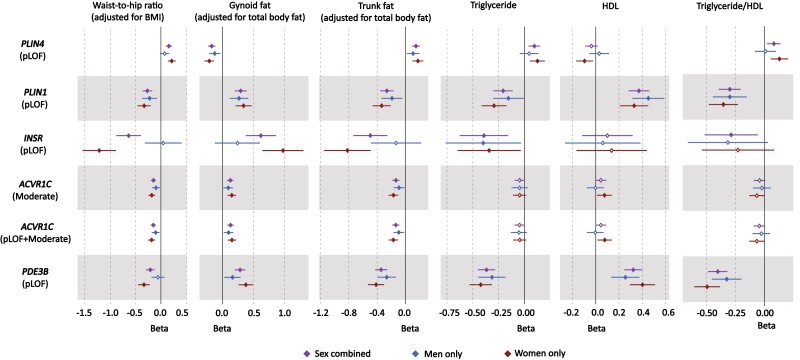
Forest plot of phenotypic associations for significant variant-gene categories. Black represents the sex-combined, blue represents the men-only, and red represents the women-only analysis. Horizontal lines represent 95% CIs. Waist-to-hip ratio adjusted for body mass index (BMI) (n = 184 246), gynoid fat adjusted for total body fat (n = 178 143), trunk fat adjusted for total body fat (n = 178 143), triglyceride levels (n = 175 271), high-density lipoprotein (HDL) cholesterol (n = 161 239), and triglyceride-to-HDL ratio (n = 161 102) were all driven from UK Biobank (See Supplementary Table 12 for details) (14).

We next assessed phenotypic associations with refined measures of fat distribution and cardiometabolic parameters and diseases. Bioelectrical impedance analysis (BIA)-derived body fat compartment measurements (17) showed that *PLIN4* (pLoF) was associated with higher android and trunk fat, and lower gynoid and leg fat (Fig. 3, Supplementary Fig. 2, and Supplementary Table 9) (14) whereas *PLIN1* (pLoF) acted in the opposite direction. Fat distribution is strongly linked to insulin resistance, but as direct indicators of insulin resistance are not currently available in the UK Biobank, we evaluated the effect of these genes on metabolic indicators typically associated with insulin resistance (18, 19) (see Fig. 3, Supplementary Fig. 2, Supplementary Table 9) (14). *PLIN4* LoF was associated with higher triglycerides (TGs), TG/HDL (triglyceride/high-density lipoprotein cholesterol) ratio, and higher glycated hemoglobin A_1c_ levels. The associations for *PLIN1* consistently contrasted with those of *PLIN4* with lower TGs, TG/HDL ratio, and additionally higher HDL cholesterol levels, in keeping with a beneficial effect on insulin sensitivity. In keeping with these findings, *PLIN4* LoF was nominally associated with an increased risk for type 2 diabetes (T2D) (odds ratio [OR] = 1.36 [1.06-1.66], *P* = .04) in the Type 2 Diabetes Knowledge Portal (T2DKP; https://t2d.hugeamp.org/), though none of the genes showed a significant association with T2D in the UK Biobank through our analysis or in the AstraZeneca PheWAS Portal (https://azphewas.com/) (Supplementary Fig. 2, Supplementary Table 9, and Supplementary Table 10) (14). *PLIN1* LoF showed nominal significance for a lower risk of cardiovascular heart disease (*P* = .03, OR = 0.55 [0.31-0.91]) in our analysis of the UK Biobank, a finding supported by an association between *PLIN1* LoF and reduced susceptibility to chronic ischemic heart disease (OR = 0.40 [0.32-0.75], *P* = 4.49 × 10^–4^) in the AstraZeneca PheWAS Portal (13).

Similarly to *PLIN1*, the combined effect of LoF variants in the *INSR* (27 variants, 61 carriers) was associated with lower WHR_adjBMI_ (β = –0.64 [–0.39 to –0.88], *P* = 6.21 × 10^–7^; see Supplementary Table 6) (14). Although a few common intronic variants (rs1035942, rs1035940, rs62124511, and rs34194998) and a low-frequency synonymous variant (rs1799815) in *INSR* have previously been associated with WHR_adjBMI_ (3, 15), the causal mechanism underlying these associations remains unknown. Our gene-based findings indicate that the *lNSR* can alter body fat distribution through LoF. Given the fact that the *INSR* gene encodes the insulin receptor itself and that both biallelic and heterozygous LoF variants in this gene have long been linked with monogenic severe insulin resistance syndromes (20), this evidence for LoF of the *INSR* having a seemingly beneficial effect on fat distribution, that is, lower WHR_adjBMI_, is surprising. Importantly, none of the *INSR* mutations previously linked to monogenic disease were present in our UK Biobank analysis. The lead *INSR* variant (p.R525X) is predicted to result in truncation of the protein within the extracellular domain preventing interaction of the extracellular and intracellular domains, and thus formation of a functional receptor. In the homozygous state, this would be expected to lead to monogenic severe insulin resistance. In terms of body fat distribution, heterozygous *INSR* LoF was also associated with higher gynoid and leg fat, and lower android and trunk fat mass (see Fig. 3, Supplementary Fig. 2, and Supplementary Table 9) (14). Similarly to the cardiometabolic associations of *PLIN1* indicating a beneficial effect, heterozygous loss of *INSR* was associated with lower TGs and a lower TG/HDL ratio (see Fig. 3, Supplementary Fig. 2, and Supplementary Table 9) (14). It was also associated with lower LDL (low-density lipoprotein) cholesterol levels but was not associated with altered HDL (see Fig. 3, Supplementary Fig. 2, and Supplementary Table 9) (14). Despite these seemingly favorable changes in fat distribution and plasma lipids, *INSR* LoF showed a nominal association for increased susceptibility to T2D in the T2DKP (OR = 3.67 [2.50-4.83], *P* = .02) (Supplementary Table 10) (14).

For *ACVR1C,* the genetic architecture of gene-based results was slightly different: Gene-based association results were statistically significant for (i) the combined burden of pLoF and moderate-impact variants and (ii) for moderate-impact variants only. There were 130 rare moderate-impact variants and 9 rare high-impact variants included in this analysis (1414 and 16 carriers, respectively). The combined effect of pLoF and moderate-impact variants and moderate-impact only variants were both associated with lower WHR_adjBMI_ (β = –0.15 [–0.10 to –0.20] and –0.15 [–0.10 to –0.20], *P* = 1.68 × 10^–7^ and 4.57 × 10^–7^, respectively; see Supplementary Table 6) (14). In this instance, the highest-ranking variant was the previously reported p.I195T variant (15, 21). In silico predictions including M-CAP (22), REVEL (23), SIFT (24), PolyPhen-2 (25), and PROVEAN (26) all assess this variant to be damaging to the protein, and structural modeling also suggests that it is likely to have a sizable effect (see Supplementary Note 3) (14). CADD (27) also estimates this variant to be among the top 1% of the deleterious variants ranked by CADD (score = 27.1).

**Figure 4.**
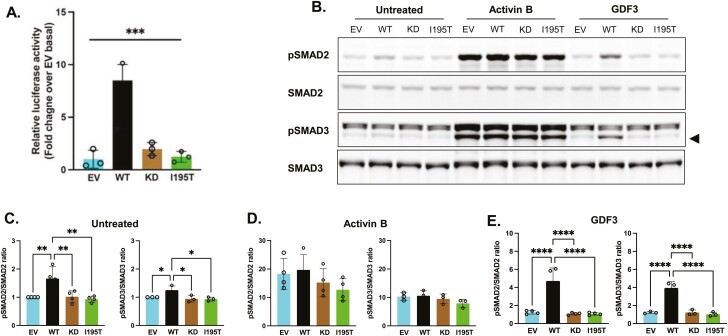
Functional impact of *ACVR1C* I195T variant on Smad signaling. A, HEK293 cells were transiently transfected with ACVR1C expression constructs and their receptor components, along with firefly and *Renilla* luciferase expression plasmids. Firefly luciferase activity was normalized to *Renilla* activity and the luciferase activity in nonstimulated cells transfected with empty vector (EV) was set to 1. A constitutively active (CA) *ACVR1C* variant T194D and a kinase-deficient (KD) variant K222R were included for comparison. Results from 3 independent experiments are presented as mean ± SD. Statistical significance was evaluated by one-way analysis of variance (ANOVA) with Tukey post hoc test for multiple comparisons between pairs. WT, wild-type ACVR1C. B to D, HEK293T cells transfected with plasmids containing EV, WT *ACVR1C*, KD *ACVR1C*, or I195T *ACVR1C* were treated for 30 minutes with activin B (25 ng/mL) or growth and differentiation factor 3 (GDF3; 500 ng/mL), and B, SMAD signaling was determined via Western blotting. Black triangle indicates pSMAD3 band. SMAD activation was quantified by the ratio of phospho-SMAD2 to total SMAD2 protein and phospho-SMAD3 to total SMAD3 protein for each condition: C, untreated; D, activin B; and E, GDF3. Data are expressed as relative pSMAD:SMAD ratios normalized to untreated EV controls. Three or 4 independent experiments were combined for analysis, and differences between ACVR1C transfection groups were determined by one-way ANOVA with Tukey post hoc test for multiple comparisons between pairs. ***P less than .001; **P less than .01; *P less than .05; ns, not significant.

To confirm the predicted deleterious effects of the ACVR1C p.I195T variant in vitro, we performed a luciferase reporter assay in HEK293 cells that strongly suggested the mutation impairs ACVR1C signaling (Fig. 4A). Similarly, while HEK293T cells transfected with wild-type ALK7 (WT) show significantly increased phosphorylation of endogenous SMAD2 and 3 when treated with both control media and media containing the ALK7 ligand growth and differentiation factor 3 (GDF3), cells transfected with kinase-deficient (KD) ACVR1C or the ACVR1C p.I195T variant fail to increase SMAD phosphorylation under these conditions (Fig. 4B, 4C, and 4E). Notably, activin B, a nonspecific activin receptor agonist, induces similar levels of SMAD2 and 3 phosphorylation in cells expressing the empty vector (EV), WT, or mutant ACVR1C constructs (Fig. 4D), suggesting that the ACVR1C p.I195T variant does not impair activin signaling globally. In sum, these in vitro experiments are consistent with the prediction that the ACVR1C p. I195T variant results in receptor loss of function.

The phenotypic associations for *ACVR1C* LoF for fat categories were similar to *PLIN1* LoF, with significant associations with higher gynoid and leg fat, and lower android and trunk fat. However, the cardiometabolic associations were less clear for *ACVR1C* with an association with lower TG and but not HDL or the TG/HDL ratio (see Fig. 3, Supplementary Fig. 2, and Supplementary Table 9) (14)).

Finally, the combined effect of LoF variants in *PDE3B* was also statistically significantly associated with lower WHR_adjBMI_ at the suggestive exome-wide threshold (*P* < 2.53 × 10^–6^) in the sex-combined analyses. However, leave-one-out analysis suggested this association was mainly driven by the premature stop variant (p.R783X, rs150090666; *P *value after dropping the variant = 0.49; Supplementary Table 11) (14). All other candidate genes remained at least nominally significant after dropping the most significant variant (see Supplementary Table 11) (14). Our analysis of the *PDE3B* p.R783X variant was in line with previous reports associating it with lower TG levels and higher HDL (28, 29). *PDE3B* p.R783X has also been reported to be associated with higher apolipoprotein B, lower apolipoprotein A1 levels, and other hematological traits (30). This variant was reported to be statistically significantly associated with a reduced risk of cardiovascular disease when meta-analyzed in the UK Biobank and other cohorts (29, 31).

## Discussion

**Figure 5.**
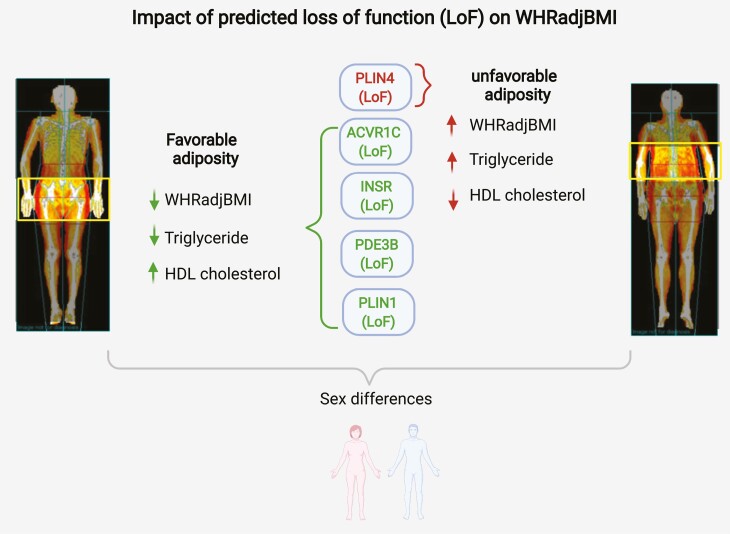
Graphical summary of the findings in this study—impact of predicted loss-of-function (LoF) of *ACVR1C*, *INSR*, *PDE3B*, *PLIN1*, and *PLIN4*.

Central adiposity has long been linked to insulin resistance and metabolic disease (32-37) but exactly why this is the case and, other than sex hormones, what exactly determines fat distribution remain incompletely understood. So, what have we learnt from human genetics thus far? First, that inheritance contributes to WHR (3, 38). Second, monogenic partial lipodystrophies indicate that single-gene variants can be sufficient to mediate substantial changes in fat distribution, classic examples being mutations in *LMNA* and *PPARG*. Interestingly, both proteins are expressed in all white adipocytes and yet specific LoF variants are consistently associated with loss of hip and leg fat whereas visceral fat is preserved (39). Third, while the beneficial effect of thiazolidinediones, one of very few drugs that clearly improve insulin sensitivity, was recognized before the discovery of *PPARG* mutations in patients with partial lipodystrophy, this link attests to the potential for human genetics to inform drug discovery. Fourth, genome-wide association studies have identified many loci associated with WHR (2, 3, 9) though these have yet to be translated into therapeutic targets. Finally, Mendelian randomization has been used to establish that genetic mechanisms linked to greater WHR_adjBMI_ can be causally linked to the risk of cardiovascular disease and T2D through either relatively *lower* gluteofemoral or *higher* abdominal fat or both (40). Furthermore, these associations are very likely to be underpinned by insulin resistance as the genetic risk score for WHR_adjBMI_ was also shown to be strongly associated with elevated fasting insulin, higher TGs, and lower HDL cholesterol (40). The data herein strongly implicate LOF in 3 genes (*PLIN1*, *PDE3B*, and *INSR*) with a beneficial effect on WHR_adjBMI_, whereas LoF of *PLIN4* appears to adversely affect WHR_adjBMI_ (Fig. 5).

Our WHR_adjBMI_ single-variant analysis in samples from 450 562 UK Biobank participants revealed missense variants in 3 genes (*CALCRL*, *PLIN1*, and *ACVR1C*) and a nonsense variant in *PDE3B*. All these genes are highly expressed in adipose tissue in keeping with emerging evidence that adiposity itself is largely centrally mediated whereas where excess energy is stored is regulated within adipose tissue itself (2, 41). *CALCRL* is also expressed in a host of other tissues, and its role in adipose tissue remains to be established (42, 43). *PLIN1* is a lipid droplet surface protein almost exclusively expressed in adipocytes and has a well-established role in regulating both TG and diacylglycerol hydrolysis (44). *PDE3B* is expressed in many tissues but has long been linked to adipocyte lipolysis, and specifically to insulin-mediated inhibition of lipolysis (45, 46). Several lines of evidence have recently implicated *ACVR1C* in the regulation of lipolysis, but it is expressed in many tissues in addition to adipose tissue and further work is required to convincingly establish exactly what it does in adipocytes (47-49).

In terms of the effect of the specific mutations present in each gene, *PDE3B* p.R783X is clearly expected to impair *PDE3B* catalytic activity, and thus potentially to increase 3′,5′-cyclic adenosine 5′-monophosphate levels and lipolysis, but the effect of the other 3 variants is far less certain (see Supplementary Note 3) (14). Interestingly, gene-based LoF burden analyses for all 4 genes were at least nominally statistically significant, suggesting that the single variants were most likely to impair function of the encoded proteins. At least when transiently transfected into cultured cells, our functional data are also consistent with the LoF predictions for the *ACVR1C* p.I195T variant.

Our exome-wide analyses confirmed statistically significant effects of LoF variants in *PLIN1*, *ACVR1C*, and *PDE3B*. In the cases of *ACVR1C* and *PDE3B*, the lead LoF variants were the same as the single variants reported previously, namely the *ACVR1C* p.I195T and *PDE3B* p.R783X variants. The fact that the phenotypic associations of the *PLIN1* p.L90P variant are directionally consistent with the *PLIN1* LoF gene burden data suggests this variant is likely to be a LoF variant too. Both the lead *PLIN1* variant and several additional *PLIN1* variants are expected to result in early truncations or nonsense-mediated RNA decay. These data are consistent with the assertion by Laver et al (50) that *PLIN1* haploinsufficiency is not associated with lipodystrophy. Interestingly, several heterozygous *PLIN1* frameshift variants had previously been linked to partial lipodystrophy (51). None of these variants overlap with those identified in the UK Biobank to date, and none are predicted to result in nonsense-mediated RNA decay. Instead, in several cases, immunoblotting of adipose tissue lysates confirmed expression of an elongated form of perilipin 1 in addition to the WT copy, so perhaps expression of these mutant forms with an altered carboxy terminus accounts for the seemingly “opposite” phenotypes (51, 52).

Gene burden testing also highlighted the role of *PLIN4* LoF variants in fat distribution, though in this case, these had an adverse effect on WHR_adjBMI_. While a higher WHR_adjBMI_ would conventionally be deemed metabolically adverse, it is possible that this need not be the case for all genetic perturbations; however, our phenotypic analyses were consistent with the predicted outcomes for all the aforementioned genes, in fact, the phenotypic associations for *PLIN1* and *PLIN4* were consistently opposite. Similar to *PLIN1*, *PLIN4* is highly expressed in adipose tissue, but it is also expressed in heart and skeletal muscle, and the *PLIN4* knockout mouse has not been reported to have an adipose tissue phenotype to date (53).

The last gene identified in the exome-wide gene burden analysis was *INSR*. In this instance, the lead variant (p.R525X) is expected to truncate the protein in the α subunit shortly before the disulfide bond normally connecting the α and β subunits. This is expected to abrogate synthesis of the functional receptor. Even if truncated protein were synthesized this would not be able to dimerize with or exert dominant negative activity over the coexpressed WT receptor, so heterozygosity for the truncating variant would be expected to reduce functional receptor protein by approximately 50%. In keeping with this, biallelic mutations in this domain usually cause extreme insulin resistance classified as Donohue or Rabson-Mendenhall syndrome. Parents of affected children have not been systematically studied and are generally held to be metabolically normal. In contrast, heterozygous *INSR* variants in the intracellular β subunit, which are synthesized and interfere with WT receptor function, cause type A insulin resistance (20). While fat mass is often reduced in Donohue syndrome, heterozygous variants associated with type A insulin resistance are not reported to be associated with fat redistribution and interestingly do not typically lead to fatty liver or dyslipidemia (20). Our data suggest, surprisingly, that the *INSR* LoF variants favorably affect WHR_adjBMI_ and LDL cholesterol. While the *INSR* LoF association with T2D was relatively weak statistically and not seen in all the cohorts assessed, it is conceivable that *INSR* LoF might adversely affect pancreatic β-cell function and/or insulin sensitivity despite the apparently beneficial effect on fat distribution. The change in TGs is somewhat reminiscent of the well-described absence of dyslipidemia in patients with monogenic severe insulin resistance due to biallelic or monoallelic *INSR* mutations, so again this does not preclude the *INSR* LOF variants being associated with reduced insulin sensitivity.

Our sex-specific analyses consistently revealed stronger effects in women than in men. These data are consistent with the fact that WHR is more strongly associated with insulin resistance in women than in men (54). Fat mass in women is consistently significantly higher than men of a similar BMI, who typically have higher lean/muscle mass. The adverse effect of a lack of lower limb/gluteofemoral fat on metabolism is strikingly apparent in patients with familial partial lipodystrophy, particularly types 2 and 3, due to specific mutations in *LMNA* and *PPARG*, respectively (39). In these and in fact in all forms of partial lipodystrophy, metabolic disease manifests considerably earlier and is typically more severe in women than in men (39, 55, 56).

Our analyses have several limitations that future work should help to resolve. First, the statistical power to detect associations, particularly when examining rare variants, depends on the sample size. Hence, there is the opportunity to discover additional findings when the WES data are released in the full UK Biobank cohort or other large-scale studies. Second, the phenotypic follow-up of cardiometabolic diseases for the candidate genes was primarily conducted in the UK Biobank, a population cohort with a limited number of cases of specific diseases. Our follow-up in T2DKP revealed the potential of data sets enriched for cases to increase statistical power in phenotypic follow-ups. Third, fat distribution is strongly associated with insulin resistance, but the UK Biobank cohort did not provide fasting samples so direct measures of insulin and inferred indices of insulin sensitivity are not available.

Our analysis strongly implicates at least 4 genes (*PLIN1*, *PDE3B*, *ACVR1C*, and *PLIN4*) in the regulation of fat distribution. The data in *PLIN1* need to be tempered by the earlier reports linking some specific *PLIN1* LoF variants with partial lipodystrophy and, the data on the *INSR* seems to suggest a potential disconnect between an apparently favorable impact of LoF variants on WHR and an apparently adverse impact on T2D risk. These findings provide valuable insight into the potential of these genes as therapeutic targets.

## Materials and Methods

### The UK Biobank Resource

The UK Biobank is a large-scale, prospective population-based study of approximately 500 000 participants aged 40 to 69 years at the time of recruitment (57). Recruitment took place between 2006 and 2010 in centers across the United Kingdom, and participants have deep phenotypic information collected from initial and repeat assessment visits, health records, self-reported survey information, linkage to death and cancer registries, urine and blood biomarkers, and other phenotypic end points. A Seca 200-cm tape was used to measure waist and hip circumference at the baseline visit, and BMI was calculated from height and weight measurements. WHR_adjBMI_ was constructed as the ratio of waist and hip circumferences adjusted for age, age (2), and BMI (measured at the baseline assessment visit). Residuals were calculated for men and women separately and then transformed using the rank-based inverse normal transformation. All additional phenotypes are described in Supplementary Table 12 (14).

### Genome-wide Association Scan of Genotyped Rare Nonsynonymous Genetic Variants

Genetic variants were genotyped in the UK Biobank using the Affymetrix UK BiLEVE or the Affymetrix UK Biobank Axiom arrays (57). Genotyping underwent QC procedures including (a) routine quality checks carried out during the process of sample retrieval, DNA extraction, and genotype calling; (b) checks and filters for genotype batch effects, plate effects, departures from Hardy Weinberg equilibrium, sex effects, array effects, and discordance across control replicates; and (c) individual and genetic variant call rate filters as previously described (57). We further excluded genetic variants with a genotype call rate below 95% and variants that were (i) not rare (MAF = 0.1%-0.5%) or (ii) not nonsynonymous or (iii) had poor correlation (*r* < 0.9) or rare allele concordance (< 0.9) when compared to the WES data (see Supplementary Table 2) (14). Genomic annotations were performed using ANNOVAR software (58). The coordinates of genotyped rare variants were lifted over from GRCh37 to GRCh38 using liftOver, and all reported positions in this study are in GRCh38. A total of 13 181 genetic variants in 7481 genes were available for analysis. Genome-wide association analyses were performed using BOLT-LMM software (59) in 450 562 participants of European Ancestry defined using a K-means clustering approach applied to the first 4 principal components calculated from genome-wide single-nucleotide variation (SNV, formerly single-nucleotide polymorphism) genotypes.

Sex-specific genome-wide association analyses were performed using BOLT-LMM software (59) in 206 082 men and 244 478 women of European ancestry from the UK Biobank. Evidence for sex differences at the variants identified in the sex-combined analysis were formally tested in unrelated individuals using a linear regression model including an interaction term between the genetic variant and sex using the same covariates used in the discovery analysis.

### Conditional Analyses and Fine-Mapping

At each associated genomic region, we conducted systematic analyses of the genomic context of associations. Our goal was to establish whether the identified rare nonsynonymous variants are likely to be the causal variants for the association with WHR_adjBMI_. At each region at 1 Mb either side of the nonsynonymous genetic variants associated with WHR_adjBMI_, we conducted both approximate and formal conditional analyses. We considered the association of all genetic variants in the regions regardless of functional annotation or allele frequency using directly genotyped and imputed data (imputed using the Haplotype Reference Consortium and UK10K haplotype resource). First, approximate conditional analyses were conducted on summary-level estimates using GCTA (60, 61) to identify sets of conditionally independent index genetic variants (*P* < 5 × 10^–8^). Individual-level genotypes for the conditionally independent variants identified in this first step were then extracted in 350 721 unrelated European ancestry participants of the UK Biobank and their independent association was confirmed in multivariable linear regression models including all variants put forward from approximate analyses. Then, at each region, we statistically decomposed the identified index signals by conditioning on the other conditionally independent index variants. We then performed Bayesian fine-mapping (62) to estimate the posterior probability of association for each variant (where 0% indicates the variant is not causal and 100% indicates the highest possible posterior probability that the variant is causal) and define the 99% credible set at that signal (ie, a set of variants in a genomic window that accounts for 99% of the posterior probability of association at that association signal). To generate credible sets, the association results at each locus were converted to Bayes factors (BFs) for each variant within the locus boundary. The posterior probability that a variant-j was causal was defined by the following:


$$\begin{array}{l} {\;\Phi \;}_{j}=\;\frac{BF_{j}}{\underset{k}{\sum }BF_{k}} \end{array}$$

where, BF_j_ denotes the BF for the j_th_ variant, and the denominator is the sum of BF_s_ for all included variants at that signal. A 99% credible set of variants was created by ranking the posterior probabilities from highest to lowest and summing them until the cumulative posterior probability exceeded 0.99 (ie, 99%).

### UK Biobank Exome-Sequence Data Processing and Quality Control

WES data of 200 643 UK Biobank participants made available in October 2020 were downloaded in VCF and PLINK formats. Details of the UK Biobank WES data processing are provided in detail elsewhere (63, 64). Further data processing and QC also has been described previously (65). In brief, we did not apply additional QC based on QUAL (variant site-level quality score, Phred scale) or AQ measures (variant site-level allele quality score reflecting evidence for each alternate allele, Phred scale). Site-level filtering was applied for targeted biallelic calls if the AB ratio (No. of alternate allele reads/total depth) was less than or equal to 0.25 or greater than or equal to 0.8. Variant-level QC filters were applied if any of the variants had (i) genotype missingness greater than 5%, (ii) maximum read depth (DP) of less than 10 across samples, or (iii) had genotype quality less than 20 for more than 20% of the calls. After applying these filters, 7.3% of the variants were flagged as poor quality and not taken forward for further analysis.

### Variant Annotation and Definition of Gene Burden Sets

We annotated variants released in the UK Biobank 200K WES VCF files in build hg38, using the Variant Effect Predictor (VEP) tool release 99 provided by Ensembl (66). In addition to default VEP features such as the consequence and effect of the variant, overlapping gene, position at complementary DNA and protein level, and codon and amino acid change, if applicable, we have used the following plugins for annotation: (i) SIFT (67), which predicts whether an amino acid substitution affects protein function based on sequence homology and the physical properties of amino acid; (ii) Polyphen-2 (25), which predicts the possible effect of an amino acid substitution on the structure and function of a human protein; (iii) CADD (27), which provides deleteriousness prediction scores for all variants based on diverse genomic features; and (iv) LOFTEE (68), which provides LoF prediction for variants. We annotated each variant using the most severe consequence across overlapping transcripts in Ensembl. We defined LOF variants as those with “high” impact predict by VEP. This includes frameshift variants, transcript ablating or transcript-amplifying variants, splice acceptor or splice donor variants, stop lost, and start gained or stop gained variants. “Moderate impact” variants include missense variants, in-frame deletion or insertions, and protein-altering variants.

### Gene-based Association Testing

In our discovery stage, we used the method STAAR (variant-Set Test for Association using Annotation infoRmation), which is a computationally scalable method for very large WES and whole-genome sequence studies and large-scale biobanks. STAAR uses a Generalized Linear Mixed Model framework that includes linear and logistic mixed models and can also account for both relatedness and population structure for quantitative and dichotomous traits (69). In our analysis, we used the genotype dosage matrix as the genotype input and covariates including age at first check (age), age (2), sex, genotyping array, top 10 genetically derived principal components (PC1-PC10) generated from the SNV array data, exome sequencing batch and the sparse GRM. For rank-based inverse normal transformed WHR_adjBMI_, we also added BMI as a covariate. We excluded the samples from our analysis if they did not pass UK Biobank QC parameters, were non-European ancestry, or if they withdrew consent from the study (n = 184 246) (65).

We ran STAAR with its default options without additional functional annotations. For each gene, with at least 2 variants with MAF less than or equal to 0.5%, we conducted gene-based association analysis for the following 3 variant categories: rare variants predicted by VEP to be a) loss of function (pLoF; ie, high impact), b) missense (moderate; ie, moderate impact), or c) both (pLoF + moderate). For each variant clustering of a gene, STAAR will provide *P* values for several collapsing burden tests including SKAT (sequence kernel association test), Burden test, and ACAT-V (set-based aggregated Cauchy association test). In addition, the output of STAAR also includes the omnibus *P* value (STAAR-O) by using the combined Cauchy association test to aggregate the association across the different tests.

After identifying the genes with STAAR-O *P* value over the threshold for exome-wide significance (*P* < 2.53 × 10^–6^), we applied more stringent QC filters on the genotype calls of the included variants. We set to missing genotype calls that did not meet the following QC criteria: 1) genotype quality greater than or equal to 20 for heterozygous variants; 2) DP greater than or equal to 7 for SNVs and DP greater than or equal to 10 for InDels; 3) a binomial test on allelic balance using the allelic depth FORMAT field for heterozygous variants with *P* greater than or equal to 1 × 10^–3^. We then repeated the STAAR analysis using the filtered genotype dosage matrix. For the gene-based analyses in this study, we used an exome-wide threshold corrected for multiple testing for 3 variant categories (*P* < 8.43 × 10^–7^) and we defined exome-wide statistical significance (*P* < 2.53 × 10^–6^) as a suggestive threshold.

To examine the extent to which the gene-based association is driven by single variants, we conducted a sensitivity leave-one-out analysis for each statistically significant gene (*P* < 2.53 × 10^–6^), testing the significance of the gene-based association after excluding each variant.

### Secondary Association Testing

We created dichotomous dummy variables using the filtered genotype dosage matrix for each identified gene, where samples with one or more rare alleles were set as “1” and the samples without rare alleles were set as “0” for different variant clustering settings of each gene. Then we combined these dummy variables into a single file and transformed it to BGEN format, which was used as the genotype input for association testing using a linear mixed model implemented in BOLT-LMM to account for cryptic population structure and relatedness (59). The GRM in BOLT-LMM was generated from the autosomal genetic variants that were common (MAF > 1%), passed QC in all 106 batches, and were present on both genotyping arrays (65). Covariates included age, sex, and PC1-PC10, genotyping chip, and exome sequencing batch. For rank-based inverse normal transformed WHR_adjBMI_, covariates also included BMI. We excluded the same group of samples as we did for STAAR analyses.

To test for heterogeneity of effect sizes between men and women for statistically significant genes identified in the gene-based analyses, we used a *Z* test to compare effect size estimates for each gene calculated in the sex-specific analyses.

### Phenotypic Associations

The gene-based phenotypic associations using the same STAAR and BOLT-LMM pipelines for the following continuous phenotypes: BMI, BIA-derived gynoid fat, BIA-derived leg fat, BIA-derived android fat, BIA-derived trunk fat, BIA-derived arm fat, TG levels, HDL cholesterol, LDL cholesterol, glycated hemoglobin A_1c_ levels (see Supplementary Table 12 for phenotype details) (14). Body fat compartments were predicted using bioimpedance measurements in the UK Biobank. The details for the prediction of body fat compartments in the UK Biobank are described elsewhere (17).

We have also investigated gene-based phenotypic associations for binary disease outcomes: T2D and cardiovascular heart disease. As BOLT-LMM is based on the linear mixed model, which cannot give an accurate effect estimate for binary variables, we have also applied a generalized linear model to estimate the odds ratio (OR) for binary phenotypes. We also looked up these binary outcomes in other resources such as the AstraZeneca PheWAS Portal (https://azphewas.com/, accessed September 2, 2021) (13) and the T2DKP (https://t2d.hugeamp.org/, accessed September 2, 2021). The AstraZeneca PheWAS Portal also uses the UK Biobank as its primary resource, but has access to a larger data set of 281 104 exomes. We looked up results for T2D (N cases = 1671; N controls = 160 949), noninsulin-dependent diabetes mellitus (defined by International Classification of Diseases, 10th Revision [ICD-10] code E11; N cases = 19 860, N controls = 182 061) and chronic ischemic heart disease (defined by ICD-10 code I25; N cases = 24 147, N controls = 176 170). In the T2DKP, we also looked up results for T2D (N = 43 125).

### ACVR1C Dual-Luciferase Assay

HEK293 cells were seeded at a density of 150 000 cells per well in 24-well tissue culture plates pretreated with poly-D-lysine. The following day, the medium was replaced with Opti-MEM I Reduced Serum medium and 550 ng of plasmid DNA; this included different pcDNA3.1-based ACVR1C constructs listed in Table 2, as well as constructs encoding receptor components (ACVR-IIB and CRIPTO) along with firefly (consisting of the SMAD-binding elements) and *Renilla* (control) luciferase reporter plasmids. Lipofectamine 3000 Reagent was used for the transfection according to the manufacturer’s protocol. Opti-MEM I Reduced Serum medium was then replaced with Dulbecco’s modified Eagle’s medium (DMEM) growth medium 6 hours post transfection.

**Table 2.** Transfected components

pcDNA3.1 construct	Receptor components
WT	ACVR-IIB, CRIPTO, pGL4.48[*luc2P*/SBE/Hygro], pRL-SV40
I195T	ACVR-IIB, CRIPTO, pGL4.48[*luc2P*/SBE/Hygro], pRL-SV40
K222R	ACVR-IIB, CRIPTO, pGL4.48[*luc2P*/SBE/Hygro], pRL-SV40
T194D	ACVR-IIB, CRIPTO, pGL4.48[*luc2P*/SBE/Hygro], pRL-SV40
EV	ACVR-IIB, CRIPTO, pGL4.48[*luc2P*/SBE/Hygro], pRL-SV40

Abbreviations: EV, empty vector; WT, wild-type.

The dual-luciferase reporter assay was performed according to the manufacturer’s protocol (Promega). Cells were washed once with Dulbecco’s phosphate-buffered saline, followed by an active lysis procedure. Briefly, 125 µL of passive lysis buffer was added to each well and the cells were subjected to one cycle of a freeze-thaw process. Cell lysates were cleared of cell debris by centrifugation at 21 130*g* for 1 minute. The assay was conducted in a 96-well plate format. In each assay, 20 µL of cleared supernatant was predispensed, followed by sequential measurement of firefly and *Renilla* luciferase using a Tecan Spark 10M plate reader (Tecan). Firefly luciferase activity was normalized for *Renilla* luciferase activity, and then further normalized with values from nonstimulated cells transfected with EV pcDNA3.1. We also studied a constitutively active (ACVR1C p.T194D) mutant and a KD (ACVR1C p.K222R) mutant for comparison (70, 71). The experiment was repeated with fresh transfections on 3 separate occasions and statistically analyzed as described in the figure legend of Figure 4.

### ACVR1C HEK293T Transfection

A total of 200 000 HEK293T (ATCC catalog No. CRL-3216, RRID:CVCL_0063; https://scicrunch.org/scicrunch/resolver/CVCL_0063) cells in DMEM High Glucose (Invitrogen catalog No. 11995065, 10% fetal bovine serum, 1% NEAA, 1% GlutaMAX, 1% Na Pyruvate, 1% antibiotics) were seeded onto to polyethylenimine-coated 12-well plates 24 hours before transfection. Cells were transfected overnight with 1-μg pcDNA3.1 plasmid DNA containing either empty vector (EV), WT human ALK7 (WT ALK7), KD ALK7 (K222R ALK7), or the I195T variant using Lipofectamine 2000 (Invitrogen catalog No. 11668030) following kit instructions.

### ACVR1C TGFB Ligand Treatment

Following transfection, cells were rinsed once and then incubated in pretreatment media (DMEM high glucose, 0.5% fetal bovine serum, 1% NEAA, 1% GlutaMAX, 1% Na Pyruvate, 1% antibiotics) for 24 hours. Following pretreatment incubation, cells were rinsed in assay media (DMEM high glucose, 0.5% FAF-BSA, 1% NEAA, 1% GlutaMAX, 1% Na Pyruvate, 1% antibiotics) then treated with or without 10 μM of the ALK4/5/7 kinase inhibitor SB431542 (EMD Millipore catalog No. 616464) for 3 hours. Dimethyl sulfoxide was used as a vehicle control in untreated cells. At the end of the 3-hour incubation, the media were removed and fresh assay media were added containing 25 ng/mL Activin B (R&D Systems catalog No. 659-AB-005), 500-ng/mL GDF3 (R&D Systems catalog No. 5754-G3-010) or ligand-free media. A total of 10-μM SB431542 or dimethyl sulfoxide vehicle was coincubated with ligands where indicated. After 30 minutes of incubation, cells were rinsed briefly in ice-cold phosphate-buffered saline and plates were flash-frozen by floating on liquid nitrogen.

### ACVR1C Western Blotting

Cells were lysed in ice-cold TNET-C Buffer containing phosphatase and protease inhibitors. Lysate was homogenized using sonication and clarified by centrifugation at 12 000*g* for 15 minutes at 4 °C. Protein concentrations were determined by BCA assay. Cell homogenate was denatured using NuPage reducing reagents (Invitrogen) and heating at 70 °C for 10 minutes. A total of 10 μg of sample was loaded on 4-12% Bis-Tris Gels (Bio-Rad) and separated at 130 V for 100 minutes using NuPage running buffer (Invitrogen) and the Bio-Rad Criterion electrophoresis system. After electrophoresis gels were incubated in ice-cold transfer buffer (Invitrogen) containing 10% methanol for 15 minutes. Proteins were transferred onto nitrocellulose membranes on ice at 100 V for 60 minutes using the Bio-Rad Criterion transfer system with plate electrodes. Following transfer, membranes were blocked for 1 hour in 5% milk, used with pSMAD3 blots, or LICOR blocking reagent (LICOR catalog No. 927-60001), for pSMAD2 blots, and probed with Abs for pSMAD2 (Cell Signaling Technology catalog No. 3108, RRID:AB_490941; https://scicrunch.org/resolver/AB_490941) and Total SMAD2 (Cell Signaling Technology catalog No. 3103, RRID:AB_490816; https://scicrunch.org/resolver/AB_490816) or pSMAD3 (Abcam catalog No. ab52903, RRID:AB_882596; https://scicrunch.org/resolver/AB_882596) and Total SMAD3 (Abcam catalog No.ab40854, RRID:AB_777979; https://scicrunch.org/scicrunch/resolver/RRID:AB_777979) overnight at 4 °C. Densities were quantified using Bio-Rad ImageLab software and SMAD2/3 activation was expressed as the ratio of pSMAD protein abundance to total SMAD protein abundance.

## Data Availability

This research was conducted using the UK Biobank resource (application Nos. 44448 and 9905). Access to the UK Biobank genotype and phenotype data is open to all approved health researchers (http://www.ukbiobank.ac.uk/).

## Abbreviations

BF: Bayes factor
BIA: bioelectrical impedance analysis
BMI: body mass index
DMEM: Dulbecco’s modified Eagle’s medium
DP: read depth
EAF: effect allele frequency
EV: empty vector
HDL: high-density lipoprotein
ICD-10: International Classification of Diseases, 10th Revision
KD: kinase deficient
LDL: low-density lipoprotein
LoF: loss of function
MAF: minor allele frequency
OR: odds ratio
pLoF: predicted loss of function
QC: quality control
T2D: type 2 diabetes
T2DKP: Type 2 Diabetes Knowledge Portal
TGs: triglycerides
VEP: Variant Effect Predictor
WES: whole-exome sequencing
WHR: waist-to-hip ratio
WHR_adjBMI_: body mass index–adjusted waist-to-hip ratio
WT: wild-type
